# “I Do Not Have to Hurt My Body Anymore”: Reproductive Chronicity and Sterilization as Ambivalent Care in Rural North India

**DOI:** 10.1111/maq.12709

**Published:** 2022-05-07

**Authors:** Eva Lukšaitė

**Affiliations:** ^1^ School of Medicine Keele University Keele United Kingdom

**Keywords:** India, care, chronicity, reproduction, sterilization

## Abstract

Drawing on 18 months of ethnographic fieldwork in rural Rajasthan, India, I examine women's narratives of chronic reproductive suffering and the practices they employed to relieve it. Cumulative effects of adverse and ordinary reproductive events and exhaustion from caregiving were often seen as reproductive suffering, while sterilization emerged as an act of care toward women's ever‐weakening bodies. Sterilization has been an integral part of the often coercive, incentive‐ and target‐driven population control program in India. Rural women, however, described sterilization not as a form of violence but as an act of care, despite its ambivalence. In the context of reproductive chronicity—a persistent reproductive suffering recurring alongside reproductive events, available care options, relations within which these options are located, and structural conditions that shape women's lives—care and suffering are intimately and ambiguously intertwined.

“*Panch minute mazaa—nau mahine sazaa*” (five minutes of pleasure—nine months of punishment) is a common saying in Chandpur (a pseudonym), a village in rural Rajasthan, India, where I did my fieldwork. People sometimes invoked this idiom in its direct form to juxtapose the short‐lived pleasure of a sexual encounter and the lengthy period of bodily suffering of a possible pregnancy. At other times, the idiom was used to denigrate activities that could be termed *mazaa*—a Hindi word for fun, pleasure, and enjoyment—and highlight dire consequences that necessarily followed in a moral world filled with prohibitions and transgressions. Rather than exploring mazaa, I focus here on *sazaa*—a Hindi word for punishment—that captures a gendered form of bodily suffering that many women I met in the village identified in their reproductive lives.

While acknowledging the joys of motherhood, my interlocutors discussed various aspects of their reproductive lives as weakening their bodies. Women across north India often describe childbearing as making them lose their strength, draining their spirit, and making them old (Jeffery et al. 1989). While nurturing and motherhood form a key part of women's lives, women have actively tried to decrease the burden that reproduction put on them (Unnithan‐Kumar 2003). Women in other contexts have similar experiences. In rural Gambia, women saw their bodies as “wearing out” due to the cumulative effects of lifetime events, particularly closely spaced pregnancies and pregnancy losses, and they tried to control the effects of these adversities through ritual, medicine, and contraception (Bledsoe 2002). The relationship between reproductive processes and women's strength is not always straightforward. Some women in south India see persevering childbirth and motherhood as giving them strength but acknowledge that biomedical interventions in birth, such as drugs to induce labor or reduce pain, interfere with this, previously unmediated, process by depleting or increasing women's power (Van Hollen 2003). My interlocutors in Rajasthan talked about the depletion of their strength, but when they did so, they did not only refer to biomedical interventions or the cumulative effects of adverse reproductive events, such as miscarriages, stillbirths, child deaths, and abortions (Das et al. 2012). Bodily complaints caused by catastrophic events merged with other, ordinary, reproductive experiences, institutional encounters, and care work.

Discourses about women's deteriorating bodies have been analyzed as ways of speaking of the structural violence that impinges on women's everyday lives. “Weakness” (*kamzori/kamjori*), in particular, has been described as a vernacular articulation of how social, economic, and political inequalities are embodied in various contexts: in a slum in Dhaka (Rashid 2007), as part of aging in South Asia (Cohen 1995), in a slum in Mumbai (Ramasubban and Rishyasringa 2001), in a psychiatric hospital in Indian‐controlled Kashmir (Varma 2020), and among Burmese women seeking abortion (Belton 2010). Women in a slum in Mumbai identified reproductive labor—the burden of housework and childbearing—as leading causes of their weakness (Ramasubban and Rishyasringa 2001). This gendered, embodied form of the chronic distress of poverty and violence presented as a combination of physical, mental, and social signs and symptoms (Belton 2010). The chronicity of bodily symptoms (Varley 2020) corresponded with the chronicity of distressing social and economic conditions.

In this article, I investigate the entanglements between chronic reproductive suffering and the acts of care women undertake to relieve it, making two related arguments. First, building on López's (2008) argument that some women see sterilization as liberation, I move the discussion on sterilization from its focus on agency and reproductive freedom to practices of care. Sterilization emerged as an act of care undertaken by some women in Chandpur to tend to their weakening bodies. Changing the conversation from decision‐making to care and shifting it to a context of rural India allows a novel perspective on sterilization to emerge. The framework of care moves beyond the question of women's agentive possibilities and offers a multi‐dimensional view of how sterilization is entangled in the everyday messiness of women's bodies, relations, and attempts to solve bodily contingencies. In India, sterilization (tubal ligation, commonly known as an *operation*)—a surgical procedure to block uterine tubes—has been a fundamental element of the often coercive, incentive‐ and target‐driven population control program. India's family planning agenda shifted its official focus between different segments of population since country's independence: it started by inviting couples to share contraceptive responsibilities in the 1950s, moved to men and vasectomies in the 1970s, and settled on women as primary contraceptive users ever since. The program often affected the most disadvantaged groups, such as the rural and urban poor, Muslims, and oppressed castes, and has been described as a form of gender violence against poor and marginalized women (Wilson 2018). Below, I describe conditions within which some rural women described sterilization not as a form of violence, but as an act of care.

Second, practices deemed as care were not in binary opposition to suffering. Care practices women employed to manage their reproductive lives—operations, temporary contraceptives, abortions, and hysterectomies—emerged as ambivalent acts that merged with reproductive suffering. Following invitations to locate care within broader values and relationships (Cook and Trundle 2020), I situate sterilization as an ambivalent act of care in the face of broader experiences of chronic reproductive suffering. Rural women experienced their bodies as chronically ill not only due to reproductive events but also due to interventions they undertook to tend to their bodies. Suffering and care intimately and ambiguously intertwined in women's experiences of what I call reproductive chronicity. Reproductive chronicity in rural India encapsulated a cycle in which women found themselves looking for relief for their recurring reproductive suffering, even as available therapeutic options, poor health care infrastructure, and fragile social and institutional relations offered temporary care. The long‐lasting, yet fluctuating nature of women's reproductive suffering that is temporarily relieved by ambivalent care options characterize reproductive chronicity.

## Care and Chronicity

In recent years, medical anthropology has taken up Mol's invitation to explore the concept of care ethnographically by focusing on its mundane, everyday practices (Mol 2008; Mol et al. 2010). The focus on care initially was seen as a move away from the anthropology of suffering to an anthropology of the good (Robbins 2013). Kleinman (2009) sees caregiving as a moral practice that makes us fully human, while Livingston (2012) describes the work of nurses, for example, cleaning cancer patients’ wounds, as deeply humanizing. Mol et al. (2010: 14) define care as “persistent tinkering in a world full of complex ambivalence.” Investigations of the politics of care problematized the view of care as an inherently moral act (Ticktin 2011) and demonstrated that while caregiving was meant to do good “it [was] also intimately bound to the possibility of disregard and evil” (Biehl 2012: 248). Whether care is embraced “as simultaneously resource and relational practice” (Buch 2015: 279) or “as a concrete work of maintenance, with ethical and affective implications, and as a vital politics in interdependent worlds” (Puig de la Bellacasa 2017: 5), what care means and what it involves depends on particular institutional, political, and social settings. Understandings of care are culturally specific, as demonstrated by Van Hollen (2018) on cancer care in south India, Świtek (2016) on Japanese eldercare by Indonesian workers, and Stevenson (2014) on the clash between Inuit and postcolonial state's understandings of care. Ambivalent dimensions of care are discussed in many ethnographic accounts—demonstrating that care is always situated within local moral worlds (Cook and Trundle 2020). Even though care often “entails risk and trade‐offs” (Seo 2020: 6), it remains central to everyday attempts to make lives more livable (Guell 2012; Han 2012; Mol 2008).

The anthropology of chronicity (Ecks 2021; Estroff 1993; Manderson and Smith‐Morris 2010; Manderson and Warren 2016; Weaver and Mendenhall 2014) continues anthropology's interest in documenting suffering (Robbins 2013). Care and chronicity are closely linked, but this link often remains implicit. In the context of chronicity—a persistent fluctuating ill health that is closely entangled with chronic changing structural conditions of people's lives—care does not simply seek to make lives more habitable but also involves endless efforts to “make suffering liveable” (Kleinman and Hall‐Clifford 2010: 249). Just as chronicity is embedded in everyday lives, political economies, biomedical regimes, and cultural systems (Estroff 1993; Manderson and Smith‐Morris 2010), so is care. A focus on the ambivalent dimensions of care highlights that care is not simply an antidote to chronicity; rather, the relationship between care and chronicity requires ethnographic attention. Both everyday caregiving practices and everyday experiences of chronicity are always locally situated, and so are their relations.

In what follows, I investigate the relationship between care and chronicity through the narratives of two of my key interlocutors whose reproductive histories correspond with experiences of many other women I came to know during fieldwork. I aim to contribute to discussions of chronicity by discussing how women's experiences of *reproductive* chronicity fill their lives with contradictory relationships that contextualize sterilization as a desired but admittedly ambivalent practice of care.

## Methods and Setting

In this article, I draw on 18 months of ethnographic fieldwork in Chandpur, a village in southern Rajasthan, India, between February 2012 and August 2013. During this time, I lived in Chandpur full time in three different locations: with a dominant‐caste family initially, then in a house rented to various meat‐eating oppressed‐caste families (meat‐eating is considered to be impure within Brahminical Hinduism), and finally in an adjacent Adivasi (indigenous) village. Each place opened distinct yet overlapping local moral worlds. While the initial introductions in the village were made by a local NGO, I built relationships by getting to know my neighbors and their kin in each location and then extended my social and trust circles. I conducted unstructured interviews with women in their homes, fields, shops, and other venues, and took part in all aspects of village life whenever I was welcome. My conversations with my interlocutors focused on women's everyday lives and reproductive experiences but involved many other topics of neighborly intimacy. Seven families became my key interlocutor families as I got to know their lives intimately, while many other people shared their stories with me during regular encounters in various village and institutional settings. I took notes in some settings and wrote detailed field notes at the end of most days. I recorded some conversations with the permission of my interlocutors. I mostly recorded interviews with people who I already knew well but I occasionally recorded conversations with people I met less frequently.

Besides village ethnography, I carried out participant observation in Chandpur's Primary Health Centre (PHC), which I attended almost daily for several months outside of the “sterilization season.” I got to know the biomedically trained staff, community health workers (CHWs), and numerous *dai‐mas* (traditional midwives). During the sterilization season, I attended sterilization camps held in two Community Health Centers, approximately 30 km away from Chandpur, where I observed interactions between biomedically trained practitioners, state functionaries, CHWs, and women who came for the procedure. I also observed women's interactions with CHWs in *anganwadis* (preschool centers), immunization camps,^1^ and outside of institutions (Luksaite 2016).

My presence in Chandpur drew curiosity, which subsided through the extensive time I spent living there. Even though I never blended in as a tall white woman, my consistent presence in village life, fluency in Hindi and increasing understanding of Mewari, and my persistence in trying things out allowed me to create trusting long‐lasting relations. Breaking hierarchies between myself and my interlocutors in institutional and village settings was the basis of my relationship‐building. Since the original fieldwork, I returned to Chandpur for two short post‐fieldwork visits in 2015 and 2016 and continue keeping in touch with some interlocutors via social media and telephone, receiving regular updates on various matters in the village. Even though the arguments I make in this article draw from a specific ethnographic moment in time, they reflect wider social and institutional relations and processes that continue to be salient to understanding rural realities in India.

Chandpur is a mixed‐caste village located in Jhadol subdistrict, Rajasthan. It serves as a center of a Chandpur zone, which is mostly populated by Adivasi villages. The nearest town and subdistrict's headquarters, Jhadol, is 25 km away and the nearest city, Udaipur, is 55 km away. Deep‐cutting social hierarchies of caste, class, and gender govern many aspects of everyday life in the area. A modest Shiva temple and a Sheetala Mata shrine under a tamarind tree mark the center of the village and the edge of bazaar, where most of village's dominant‐caste residents live. Life in the village is precarious for most residents, except for a few wealthy and well‐connected households. Wealth and health are somewhat distributed alongside the lines of intersecting hierarchies. Most Adivasi and Dalit households rely on agricultural and daily wage labor and live in chronic poverty and poor health. Dominant‐caste families often hold stable income from government jobs and access private hospitals in Udaipur for health care. Most other families fall somewhere in the middle and rely on irregular income and agricultural labor and have various health trajectories.

During the period of my research, the average daily income for manual laborers in the construction sites in Jhadol ranged from Rs.70 (US $0.93) for carrying and sieving stones, usually done by women, to Rs.300 (US $4) for a supervisory role and mixing cement. The rates were higher in Udaipur, but daily commuting costs added a considerable expense. Most households grew corn, wheat, and lentils for their own consumption, while vegetables grew only in the fields of families who could afford irrigation. Even though most people owned their houses and consumed produce from their fields, extra costs associated with kinship obligations were high and frequent.

While the quality of public health care in rural India improved with the introduction of the National Rural Health Mission in 2005 (Gopalakrishnan and Immanuel 2017), the bleak picture that existed before (Banerjee et al. 2004) did not disappear. Health and health care provision in rural Rajasthan remains poor. Public health infrastructure in rural Rajasthan focuses on maternal and infant health, and most government interventions target women of reproductive age and seek to govern various aspects of their lives through a network of biomedical and state institutions. While CHWs—auxiliary nurse midwives, accredited social health activists (ASHAs), and anganwadi workers—provide access to nutritional supplements, contraception, pregnancy care, and operations, key government plans, such as Janani Suraksha Yojana, a Safe Motherhood Initiative, promote institutional deliveries. Medical pluralism characterizes health‐seeking behavior and traditional practitioners not only continue to provide maternity care outside institutions (Roy et al. 2021) but also carve out space within institutional deliveries in often unexpected ways (Lukšaitė 2021). Numerous private practitioners with different levels and systems of training have set up “medical shops” in Chandpur and provide diagnosis and treatment. Abortions in Rajasthan are relatively common (Ahmad et al. 2020), but inadequate quality and provision within the public sector means that women continue to rely on untrained providers (Iyengar and Danielsson 2018) despite recent legislation to improve access.^2^

Management of reproduction has been a preoccupation of the Indian government since the country's independence. In the name of national development, increasingly coercive population control measures were undertaken at the recommendation of the World Bank, the Ford Foundation, the Population Council, and other international organizations (Connelly 2006; Mamdani 1972). The national population control program moved from advocating a rhythm method in early 1950s (Ledbetter 1984) to a “cafeteria approach” and the introduction of targets and incentives in the mid‐1960s (Satia and Maru 1986) to forced vasectomies in the 1970s. The National Emergency in the 1970s is still remembered as “*nasbandi ka vaqt*” (a time of vasectomies) (Tarlo 2003), when a reported eight million people—mostly illiterate, economically and politically disadvantaged men—were forcefully sterilized. The memories of the Emergency and the development of laparoscopic surgery techniques contributed to tubal ligation becoming one of the most prevalent methods of contraception by the 1980s. With the return of programs focusing on women as preferred users of family planning (Basu 1985), “(t)he pressure to meet quotas, the obsession with efficiency, and the urgency to defuse India's population bomb” cemented the reliance on laparoscopic sterilizations (Olszynko‐Gryn 2014: 164). Discourses on reproductive health and rights that proliferated in the 1990s continued to suggest reducing poverty and population growth by controlling women's fertility (Qadeer 1998). Tubal ligation continues to be the most prevalent method of contraception, especially among the rural and urban poor. In 2015–16, 36% of married women aged 15–49 used tubal ligation, half of them before the age of 26 (IIPS and ICF 2017: 111–13).

In a context where poor women's fertility has been politicized as an obstacle to India's economic development (Wilson 2018), and where the lack of quality of care in sterilization camps led to numerous women's deaths (Sharma 2014), my interlocutors’ narratives illustrate how they see sterilization as an act of care toward their weakening bodies. This care merges with other factors to produce reproductive chronicity.

## Tiya's Chutti: Sterilization as an Act of Care

To get to Tiya's mud house on the top of the hill in a scattered Adivasi village, one must catch a jeep from Chandpur or walk for 3 km on a windy asphalt road. Tiya was in her early 40s and a mother of three teens, two boys and a girl. Her husband worked irregular and low‐paid jobs on construction sites around Chandpur, in Udaipur, and other parts of Rajasthan and Gujarat, while Tiya looked after their children, the house, kinship responsibilities, and the fields. Tiya said that whatever she grew in their fields got them through the year: If^2019^). Tiya's daughter returned after one season on the cotton farm and refused to go back. When there was no cash in the house, Tiya worked on National Rural Employment Guarantee Act (NREGA)^3^ projects around Chandpur earning between Rs.70–120 (0.93–1.60 $) for a full day's work.

Tiya told me that besides her three surviving children, she had another boy, Nitin, who would have been around 12 at the time of our conversation. He died in the arms of his father, when he was around seven, from an undiagnosed illness. Tiya told me that they decided to stop having children after Nitin, their third child, was born, but did not get the procedure organized in time and she became pregnant with the fourth child. She sought advice from a *bhopa* (local healer) on how to induce abortion and was given herbal medicine. Pharmaceutical abortion drugs—a combination of mifepristone and misoprostol—were not widely available in the area 10 years ago. The child did not “fall,” and she carried to term. Several months after the birth of another son, a local anganwadi worker approached Tiya and took her to the government‐organized sterilization camp to get her tubes tied.

When I met Tiya that day, she had just visited the Chandpur market to get household essentials: soap, oil, and sugar. She dropped by the house of another Adivasi woman, Paru. Paru, in her mid‐20s, had an arranged marriage in her late teens and was the mother of three daughters and one son. Her husband ran a tea stall in the bazaar but spent most of his income on alcohol. Paru looked after her children, the fields, and around 15 goats. That day, Paru's mother‐in‐law, who lived in a house nearby, met Tiya and me at the courtyard of Paru's mud house and said that Paru was in bed and in pain because she took the *MCwalli* pill. “MC” was a common way women referred to menstruation, an abbreviation of an English term “monthly cycle” (Rajagopal and Mathur 2017). The phrase “MCwalli pill” referred to pharmaceutical abortion drugs—a combination of mifepristone and misoprostol—increasingly available in rural India (Ramachandar and Pelto 2005). Paru's mother‐in‐law said they received it from a nurse, but it remained unclear which health care worker she had in mind. Later, Paru shared that she did not have time to get the sterilization operation organized, but neither she nor her husband wanted another child, as they were already struggling to feed their four existing children. That day at Paru's courtyard, Tiya said without hesitation: “That is why I had the operation, now it is *chutti*—I do not have such problems anymore. I do not have to hurt my body anymore.”

Chutti literally means “holiday” or “vacation,” a break from school or household chores, but it can also mean a termination of something. One can get chutti from marriage (divorce) or from work (getting fired). There was a sense of relief in Tiya's use of chutti to describe ending childbearing; it also ended the bodily suffering that seemed inevitable in a woman's life. This was not the only time Tiya used chutti to describe herself as a woman of post‐childbearing age. A few months before, I entered Tiya's kitchen while she was making *rotis* (flatbreads) seated on the floor next to the fire. We had talked about her operation before, and I remembered the ease with which she described it: “I had four children and then I had the operation and then chutti. Four is enough. My family is happy, so I am happy too.” Tiya's chutti came later than suggested by decades of family planning campaigns and slogans, such as “Hum Do, Hamare Do” (we two, our two), which advocated two children as an ideal number. The small family that existed in bureaucratic imaginaries gained different forms when it “trickled‐down” to villages (Sarcar 2020), and it was often defined by sterilization, not the number of children (Patel 2007). The program's messages penetrated the most remote villages through its publicity campaigns (Connelly 2006). Tiya rephrased a common slogan, “A small family is a happy family.” Tiya's relief referred to ending childbearing and bodily suffering while complying with the state's ideas of appropriate motherhood, demonstrated by invoking a family planning slogan.

Like the other women I spent time with in Chandpur, Tiya experienced her body as unpredictable due to uncertain access to contraception, unexpected pregnancies, and unreliable availability of medical abortion. Reproductive events disrupted women's bodies and everyday lives. This unpredictability and disruption resonates with findings in anthropological work on chronic illness, in which chronically ill bodies are often described as volatile, with symptoms manifesting at unpredictable times (Toller and Farrimond 2021; Wendell 2001). My interlocutors saw being a woman in reproductive years as an affliction—a cause of persistent and unpredictable suffering. Unintended pregnancies and terminations were seen as flare‐ups that contributed to bodily deterioration and required therapeutic interventions. Even though their chronic illness experience did not emanate from a pathology identified and localized through a biomedical paradigm, it incorporated many characteristics that a biomedical approach attributes to chronic illness: persistence, unpredictability, disruption, and the focus on self‐care and management. Tiya, like many of her neighbors, undertook sterilization to relieve chronic reproductive suffering. Chutti, and Tiya's words “I do not have to hurt my body anymore,” articulated therapeutic relief.

When I asked Tiya if she was scared before the operation, she said that she was, and added, “It took as long as smoking a *bidi* [thin hand‐rolled cigarettes]. You know, you light the bidi, it takes three minutes, and it is over. That is how long the operation took; I did not even realize, and it was over.” Tiya's words inverted the idiom I used at the beginning of this article (“five minutes of pleasure—nine months of punishment”). She endured a moment of pain for a lifetime of relief. While cure is often contrasted to care in the juxtaposition between acute and chronic illness, this distinction has been questioned by medical anthropologists (Manderson and Smith‐Morris 2010), particularly because it fails to capture bodily realities in contexts of chronic poverty and ill health, where therapeutic interventions often focus on symptom relief rather than addressing underlying causes (McDowell 2017).

Working with Puerto Rican women in New York, López (2008) argued that women chose to end their reproductive lives by sterilization. Sterilization was a decision they made in response to their social and economic conditions, and they saw that decision as liberating. Many poor women across India, too, saw the operation as a positive experience contributing to their emotional health (Brault et al. 2016; Säävälä 1999). I suggest that the framework of care allows us to move beyond more common discussions on agency, victimhood, and reproductive freedom by focusing instead on tensions, uncertainties, and contingencies. While frameworks investigating reproductive decision‐making and agentive possibilities provide a useful lens for understanding women's reproductive experiences, they often fail to capture the everyday messiness of bodies entangled within relations and women's desperate, hopeful, and pragmatic attempts to solve emerging bodily problems. Cook and Trundle's (2020: 180) idea of “unsettled care” allows us to investigate a “range of hopeful, doubtful, or ambivalent attitudes to and experiences of care, even in contexts of structural disenfranchisement.” This approach to care places the focus on women's complex embodied experiences of navigating reproduction and life in rural India rather than on neat, linear questions on their agentive possibilities. While I have already discussed the hopeful possibilities of sterilization as care, the narrative that follows highlights its more ambivalent dimensions.

## Bindu: The Continuum of Suffering and Care

Bindu's house was located on the outskirts of Chandpur alongside other Dalit houses. Unlike most Dalit families, Bindu's family had a brick house and was economically secure. Her husband ran a small painting business in a nearby town, and Bindu, who was in her 30s, sold vegetables in the bazaar. Bindu had three children—a daughter and two sons—who attended private schools in the village, marking their parents’ aspirations to social mobility and increased wealth (Chavan 2013). When her youngest child was five, Bindu wanted to end childbearing and was given pills that were meant to “close” her uterus. But the pills did not work, and she got pregnant again. She went to Udaipur for an abortion because, she said, “Three are enough, it is difficult enough to take care of them; who wants to run after another young one?” She then underwent sterilization, eight years before our conversation in 2013.

That day, as we settled in her bedroom, Bindu insisted that I record her. We had known each other for a year by then but I had only taken notes during or after our conversations. Bindu told me about her experiences of childbirth, childrearing, contraception, and relationships with her husband and in‐laws. I asked her if she thought there was any taboo in discussing the operation in Chandpur and why many people, in response to learning about my research, pointed to the houses in the distance where women who had their tubes tied lived, saying: “Magna Devi had it some years ago, Kanku had it last month.” Bindu confirmed that people often knew if their neighbors or kin had the procedure and shared this information without hesitation:

Nowadays it is easy to tell your neighbors, friends, or doctors that you had the operation. This topic became open. Before, women used to feel ashamed to speak to male doctors about pregnancies or abortions, but not nowadays. In those days, they used to fully cover their faces and would not even wear shoes. When I got married, I did not wear my slippers in front of my in‐laws also. I used to carry slippers in my hand all the way until that house [she pointed to the last house in her lane] and only then would wear them to go to the market. I did not speak to my father‐in‐law directly at all; if I needed something from him, I had to ask somebody else. But then it all changed.

Bindu spoke of the shame that used to be associated with pregnancies, abortions, and sterilizations and drew parallels with strict rules of behavior for daughters‐in‐law within their in‐laws’ households prevalent across north India (Jeffery et al. 1989). Looking back, Bindu highlighted the practice of *ghunghat*—women covering their heads or faces with loose ends of their saris in the presence of their husbands’ male relatives, which can extend to being unseen or unheard—as an example of changing practices. The need to remain unheard directed daughters‐in‐law not to address their fathers‐in‐law directly while making sure that even the sounds that their slippers made remained unheard (Abraham 2010). Ghunghat and remaining barefoot in the presence of male kin were embodied hierarchies that Bindu herself had to enact. The social change that removed strict gendered rules of conduct within the home and the shame that mediated reproductive encounters remained vaguely timed in Bindu's narrative, but other ethnographic accounts also report women's experiences of fundamental social transformations that affected how they perceived their own health, strength, and power alongside reproductive processes more generally (e.g., Van Hollen 2003). Bindu affirmed the ease with which women discussed their operations with neighbors, kin, and health care professionals nowadays.

Bindu's operation eight years before we spoke must have happened after this social change took place. She said that her husband forbade her to get the operation: “But I said I need it no matter what you say. If I die, just burn me but I am getting the operation because I do not need any more children, I told him. He did not even come with me to the hospital.” Bindu's parents were against her operation, too. They were afraid that something might happen to her. Her in‐laws were worried that she already looked weak and would get even weaker after the operation. Bindu, like Tiya, saw sterilization as a necessary therapeutic intervention that she had to undertake, even against her household's approval.

Bindu elaborated on why tubal ligation, for her, was an act of care by explaining the various challenges that reproductive processes and their management posed. Just as for Tiya, abortion emerged as the defining illustration of women's reproductive suffering: “If one gets pregnant, it does not ‘fall’ that easily. For some women the abortion pill works, but for me it never does, no matter which or how many pills I take.” She spoke about her fertility management:

And if you forgot to eat the [contraceptive] tablet, you need to go to the doctor every time and pay him money and your body suffers every time. Why would you do that? Just close it for good. I had my children, didn't need more and this is why I got an operation. Men's bodies do not get weak, it's our bodies which deteriorate. How long can we run around with children on our hands; how long can we wash their dirty clothes? I have never seen more blood than when I got an abortion; I do not want to go through anything like that again. That is why I got an operation. And now my children got big, now we are waiting for the time to arrange their marriages.

Bindu spoke about bodily deterioration caused by reproductive events and technologies. She spoke of abortion as simultaneously a relief and a cause of suffering. While providing a solution to terminate an unwanted pregnancy, it further weakened women's bodies through blood loss. She saw reliance on biomedical institutions and technologies as simultaneously part of her pragmatic access to desired outcomes and a contribution to her suffering. Going to the doctor *every time* was burdensome. The loss of blood and money impinged on relationships of dependence—Bindu's on doctors and her children's on her—relationships that were simultaneously desirable in some contexts and burdensome in others. Bindu spoke of her love for her children alongside her exhaustion from caregiving.

In Bindu's narrative, sterilization emerged as part of everyday reproductive care practices that women employed to manage contingencies in their reproductive lives. While all women actively engaged in self‐care practices to make their bodies workable (Guell 2012) and to find “more bearable ways of living in—or with—reality” (Mol 2008: 46), women's perceptions of what constituted such care and how it interrelated with suffering differed. Self‐care was something that was necessary in the context of resource‐poor health sector and the gendered division of reproductive labour within the household, but it was also something that could be easily redefined by the family planning discourses (Sarcar 2020) or contraceptive advertisements constructing women's needs and providing solutions (Appleton 2019).

Bindu spoke about operation, contraception, and abortions as ambivalent practices. For Bindu and Tiya, sterilization stopped bodily deterioration, but Bindu's in‐laws thought it inflicted weakness. Many women in Chandpur spoke about kamzori: tiring quickly and being unable to walk long distances or carry water from far away pumps or wood from the forest for food preparation. Some wanted the operation to stop weakness, while others believed that the operation caused weakness (Ramasubban and Rishyasringa 2001). Weakness, as a form of chronicity, filled women's lives with contradictory causal relationships.

Bindu narrated her endless efforts at trying out different pills—for closing her uterus, for making the fetus fall, and for daily contraceptive consumption—and how they did not work or were difficult to obtain or consume. Social relations that provide access to care are fundamentally important, but rural women wanted to avoid their dependence on temporary contraception and on the CHWs who supplied them. Other women, too, were reluctant to consume contraceptive pills. Bindu's neighbor dismissed contraceptive pills because she preferred injections over pills generally (Pinto 2004). Other women were concerned with the side effects of a daily consumption of pills (“if you have to eat it every day, it cannot be good for you”), while women who lived with in‐laws often felt unable to safely store and consume contraceptive pills, something that resonates with findings in other contexts (Appleton 2020). The permanency of the operation was desirable precisely because it brought a relational finitude and independence (Trundle 2020).

Rural women saw sterilization as an act of self‐care *despite* its ambivalence. Women who had the operation with or without the support of their kin did so to tend to their bodies within available care regimes without submitting to or resisting narratives of victimhood or backwardness.

## Conclusion

While Tiya and Bindu wanted tubal ligation operation to be a permanent relief, some older women's narratives described the operation as ineffective in the long term. Many older women in Chandpur experienced heavy bleeding or pain after sterilization and, consequently, underwent hysterectomies. Women framed their hysterectomies similarly to sterilizations, as interventions to end bodily suffering and minimize future risk. This corresponded with other ethnographic accounts. For instance, Desai (2016: 14) investigates why rural low‐income women in a neighboring state of Gujarat undergo hysterectomies at an average age of 36 and quotes one of her interlocutors: “After the operation [hysterectomy], I knew I will have my body back. I knew that my health would not suffer anymore. I would have no more worries.” Two‐thirds of Desai's informants have undergone tubal ligations about 10 years prior to their hysterectomies. Older women in Chandpur, too, noted that sterilization was only a temporary solution, and that permanent relief was provided by hysterectomies. But hysterectomies were associated with the early‐onset menopausal symptoms. The desire for definitive cures gave way to a continuum of care and chronicity: operations relieved suffering temporarily but various symptoms recurred requiring regular tinkering (Mol et al. 2010).

Sterilization is an integral part of population control programs that reproduce caste, class, gender, and geographical inequalities by targeting the most marginalized sections of India's population, and hysterectomies are often performed on rural women without medical necessity and through predatory hospitals (Xavier et al. 2016). Women considered both interventions as acts of care for their weakening reproductive bodies. While services and practices deemed by some women as care may not look like care from other women's perspectives, the “critical ethnographic respect” framework (Appleton 2020) allows us to investigate the multiple meanings of care and how they are constituted in different contexts. Listening to women's experiences of care alongside narratives of everyday life demands, while layering them with an analysis of structural conditions, allows us to understand how social and material conditions situate women's experiences. What constitutes care for different women in contexts of chronic poverty and ill health is affected by what services, information, and relations are available.

My interlocutors’ accounts of reproductive suffering and therapeutic efforts undertaken in tandem with partners, kin, and institutions merged into what I call reproductive chronicity: a form of persistent, yet fluctuating, bodily suffering that is temporarily relieved by ambivalent care options but recurs alongside reproductive events, relations within which care options are located, and structural conditions that shape women's lives. Discussing multimorbidity in a deprived area of the United Kingdom, Ecks (2021: 520) describes “the chronicity of both the problems and of the treatments” and how rather than providing relief, interacting side effects of multiple medications, the lack of follow‐ups or explanations, economic precarity, and social dysfunction can deepen chronic physical and mental conditions. Reproductive chronicity, similarly, captures not only the cumulative effects of catastrophic and ordinary reproductive processes but also available solutions and the efforts required to access them. Women had to mobilize their intimate and social connections to achieve care, but these connections were often unreliable. Reproductive chronicity connected the “failures of the body to failures of one's social world” (Das 2015: 32), and it made sterilization a desired but ambivalent practice. Precisely because women's therapeutic practices continuously failed to provide a cure within a resource‐poor, hierarchy‐rich, and population control–focused health care system, practices of care intertwined with bodily suffering in a never‐ending cycle.

## Funding information

This article is based on fieldwork that was funded by the Brunel University London PhD studentship, the Parkes Foundation PhD grant, and the Royal Anthropological Institute's Sutasoma Award.

## Notes

1 Udaipur and Jhadol Chief Medical and Health Offices and Marie Stopes India granted permissions to conduct fieldwork in sterilization camps. The permissions to access PHC and anganwadis were given by staff. While explicit permissions and invitations by staff were paramount in gaining access to institutional settings, I sought explicit permissions from women and their companions attending these spaces in every encounter.

2 The Medical Termination of Pregnancy (Amendment) Act 2021 reduced the number of registered medical practitioners required to obtain the pills, but pharmacists in Rajasthan rarely stocked these drugs due to legal barriers and excessive documentation required (Chandrashekar et al. 2019).

3 NREGA is an Indian labor law aiming to provide at least 100 days of guaranteed wage employment in a financial year to every household whose adult members volunteer to do unskilled manual work.
